# SPECTRA: a tool for enhanced brain wave signal recognition

**DOI:** 10.1186/s12859-021-04091-x

**Published:** 2021-06-02

**Authors:** Shiu Kumar, Tatsuhiko Tsunoda, Alok Sharma

**Affiliations:** 1 School of Electrical and Electronics Engineering, Fiji National University, Suva, Fiji; 2 Laboratory for Medical Science Mathematics, RIKEN Center for Integrative Medical Sciences, Yokohama, 230-0045 Japan; 3 Department of Medical Science Mathematics, Medical Research Institute, Tokyo Medical and Dental University (TMDU), Tokyo, 113-8510 Japan; 4 Laboratory for Medical Science Mathematics, Department of Biological Sciences, Graduate School of Science, University of Tokyo, Tokyo, 113-0033 Japan; 5 School of Engineering and Physics, The University of the South Pacific, Suva, Fiji; 6 Institute for Integrated and Intelligent Systems, Griffith University, Nathan, Brisbane, QLD Australia

**Keywords:** Brain computer interface (BCI), Common spatial pattern (CSP), Common spatio-spectral pattern (CSSP), Electroencephalography (EEG), Motor imagery (MI), Tangent space mapping (TSM)

## Abstract

**Background:**

Brain wave signal recognition has gained increased attention in neuro-rehabilitation applications. This has driven the development of brain–computer interface (BCI) systems. Brain wave signals are acquired using electroencephalography (EEG) sensors, processed and decoded to identify the category to which the signal belongs. Once the signal category is determined, it can be used to control external devices. However, the success of such a system essentially relies on significant feature extraction and classification algorithms. One of the commonly used feature extraction technique for BCI systems is common spatial pattern (CSP).

**Results:**

The performance of the proposed spatial-frequency-temporal feature extraction (SPECTRA) predictor is analysed using three public benchmark datasets. Our proposed predictor outperformed other competing methods achieving lowest average error rates of 8.55%, 17.90% and 20.26%, and highest average kappa coefficient values of 0.829, 0.643 and 0.595 for BCI Competition III dataset IVa, BCI Competition IV dataset I and BCI Competition IV dataset IIb, respectively.

**Conclusions:**

Our proposed SPECTRA predictor effectively finds features that are more separable and shows improvement in brain wave signal recognition that can be instrumental in developing improved real-time BCI systems that are computationally efficient.

## Introduction

Brain–computer interface (BCI) is one of the emerging technologies for neuro-rehabilitation that offers paralyzed people a non-muscular channel of control and communication to the external world [1, 2]. Complex and unique brain wave patterns are generated for each different brain activity and it is very difficult to manually decode and identify the different categories of these brain wave signals. Therefore, many research work [3–9] are being carried out to automatically identify the different categories of the brain wave signals with high accuracy as it is useful in many applications such as seizure detection [10, 11], sleep stage recognition [12], emotion or stress recognition [13, 14], neuro-rehabilitation [15, 16], and gaming [17, 18].

Non-invasive electroencephalography (EEG) sensors are usually placed around the scalp to capture the brain waves generated by the brain activities. The EEG signals can then be mapped to various commands for controlling external devices after a chain of refined signal processing and machine learning procedures such as filtering, feature extraction and classification of the EEG signal. Thus, deliberately generating different brain wave patterns will enable individuals to control external devices.

P300 [1, 19–21] and motor imagery (MI) [18, 22–25] are the two methods for obtaining the EEG signal for EEG-based BCI systems. In P300, usually the parietal and occipital areas are used to obtain the distinctive EEG signals 300 ms after the visual stimulus. This study focuses on the MI-based BCI that uses the sensorimotor rhythms. The Mu and Beta rhythms are recorded from the sensorimotor cortex region of the scalp producing different patterns for different MI tasks, which can be processed and used for BCI control.

In MI-based BCI systems there are several problems that need to be taken care of such as the pre-processing algorithm for noise removal/reduction, selection of the frequency band(s), feature extraction and classification. Filtering is usually applied as the pre-processing algorithm. With a vast range of filtering methods available, common average filtering [26, 27], Laplacian filtering [28, 29] and FIR bandpass filtering [30] are the most commonly used filtering methods. A number of feature extraction methods [31–43] and classification algorithms [23, 32, 44, 45] have been proposed due to the fact that the reliability and feasibility of MI-based BCI systems largely depend on robust and effective feature extraction and classification of EEG signals. Common spatial pattern (CSP) [9, 23, 36, 37, 39, 41–43, 46–55] has been widely used for feature extraction of EEG signals for MI-based BCIs. The selection of frequency bands plays a major role in extracting significant CSP features from MI EEG signals. The optimal frequency bands are generally subject-dependent and manually tuning the frequency band is a challenging and tedious task. To tackle this problem, various frequency band selection approaches have been proposed [9, 38, 50, 56–61]. Novi et al. [59] proposed sub-band common spatial pattern (SBCSP) approach. In their approach, EEG signals are decomposed into multiple non-overlapping sub-bands and the CSP features extracted from each sub-band are fused together and used for classification. Filter bank CSP (FBCSP) was proposed by Ang et al. [56], which uses multiple overlapping sub-bands for decomposing the EEG signals into a number of sub-bands. The CSP features obtained from these sub-bands are then fused together and feature selection is employed for selecting important features. To improve the FBCSP approach, a discriminative FBCSP (DFBCSP) [61] approach was proposed. It utilizes Fishers ratio for choosing the significant subject-dependent sub-bands. Wei and Wei [38] proposed a binary particle swarm optimization method for selecting significant sub-bands from a set of pre-determined sub-bands.

A sparse filter bank CSP (SFBCSP) [62] approach, which utilized multiple sub-bands for optimizing the sparse patterns has also been proposed. Sparse Bayesian learning is increasingly gaining widespread attention and used for various purposes such as feature selection [42] and classification [63]. Zhang et al. [42] proposed a sparse Bayesian learning of filter bank (SBLFB) approach in which sparse Bayesian learning is used for automatically selecting the significant features. A spatial-frequency-temporal optimized feature sparse representation based classification (SFTOFSRC) [36] has been proposed with a focus of optimizing CSP features in subject adapted space-frequency-time patterns.

In this work, we mainly focus on the feature extraction process. Feature extraction is one of the essential steps in machine learning and signal processing having a vast impact on the performance of the algorithms in these fields. The extraction of significant features is essential as selecting redundant or insignificant features will degrade the performance of the system. This work extends our previous work on CSP-TSM (tangent space mapping) [64] approach. In the CSP-TSM approach, a single window is used to extract the CSP and TSM features followed by feature selection using least absolute shrinkage and selection operator (Lasso). In this paper, we propose to use multiple temporal delayed windows to extract features that are more separable. Using multiple windows give rise to problems such as the window size and number of windows to use. Therefore, these problems are also addressed in this work. Furthermore, we take advantage of the common spatio-spectral pattern (CSSP) approach. In CSSP, a temporal time delayed signal is inserted to the raw signal. The value of time delay $\tau$ used also influences the performance of the CSSP algorithm. The problem of selecting the appropriate $\tau$ value is also addressed in this work. Thus, this work combines the CSP-TSM and CSSP approaches to take advantage of both approaches which boosts the performance of the overall system.

The TSM approach uses Riemannian distance to Riemannian mean which provides superior information about the class membership compared to the CSP approach that utilises Euclidean distance to its mean. On the other hand, the CSSP approach improves the spatial resolution of the signal. Therefore, taking advantage of these approaches i.e. appropriately combining CSP-TSM and CSSP approaches should yield features that are more effective and significant in classifying the MI EEG signals. To validate and compare our approach with other competing methods, public benchmark datasets: BCI Competition III dataset IVa, BCI Competition IV dataset I and BCI Competition IV dataset IIb have been used. The proposed scheme successfully extracts more significant features which accounts for the reduced error rates achieved (refer to results section) for all three datasets. Promising results are obtained, thus the proposed scheme can play a key role in developing improved MI-based BCI systems.

The main contributions of this work are as follows:

- We have combined CSSP with the CSP-TSM approach resulting in CSSP-TSM. TSM is retained as it gives superior information about the class membership while the use of CSSP improves the spatial resolution of the signal and thus further boosts the overall performance of the system.
- Use of CSSP involves inserting a temporal delayed window to the trial signal. Therefore, we have proposed the use of multiple overlapping temporal windows for extracting more significant features. We have addressed the problem of the number of multiple windows to use with the proposed scheme and how the multiple windows obtained can be combined to result in CSP-TSM and CSSP-TSM approaches for improved performance. Also, the time delay $\tau$ used influences the performance of the system and varies among different subjects. Therefore, a cross-validation approach has been proposed for the selection of time delay $\tau$ in order to obtain optimal performance for each of the subjects.
- Several feature selection methods have been evaluated to determine which feature selection method is best for selecting significant features. Use of F-score showed superior performance in selecting the significant features over other feature selection methods (Lasso—used in original CSP-TSM approach, mutual information, and sparse Bayesian learning). Thus, F-score is recommended for feature selection over other methods that have been evaluated and have been used in this work.

## Results

The processing in this work has been carried out using Matlab. Moreover, all training and test have been performed using each subject’s data, i.e. the data from other subjects are not used. In this study, the MI EEG data between 0.5 and 2.5 s (i.e. 200 sample points for dataset 1 and 2, and 500 sample points for dataset 3) after the visual cue have been extracted and used for further processing to obtain the results of all the competing methods. Common average referencing has been used as the pre-processing step for each individual EEG trial. A Butterworth bandpass filter has been used for filtering and classification is done using the SVM classifier (which is trained using training data) for all the methods. A 7–30 Hz wide band have been used for the conventional CSP approach. To make a fair comparison six spatial filters have been used for all the methods while keeping all other parameter settings the same as proposed by the reported works. The performance of all the experiments conducted have been evaluated using 10 × tenfold cross validation. The values after the ± sign in Tables 1, 2, 3 represent the standard deviation.

**Table 1:** Error rates (%) of proposed SPECTRA predictor and competing methods for dataset 1

Subject	CSP	CSSP	FBCSP	DFBCSP	SFBCSP	SBLFB	CSP-TSM	SPECTRA (Proposed)
*aa*	21.00 ± 5.31	17.00 ± 7.34	17.14 ± 8.19	**9.65** ** ± 5.01**	18.43 ± 7.45	16.79 ± 8.93	16.79 ± 6.29	10.36 ± 6.10
*al*	3.86 ± 3.63	3.07 ± 3.03	1.29 ± 1.18	**1.00** ** ± 1.91**	1.64 ± 1.36	1.36 ± 1.23	2.14 ± 3.53	1.07 ± 2.51
*av*	28.29 ± 7.46	28.86 ± 7.10	30.36 ± 8.23	31.21 ± 8.92	29.93 ± 6.44	28.07 ± 8.45	24.90 ± 9.10	**21.67** ** ± 5.93**
*aw*	10.36 ± 5.10	8.43 ± 5.09	6.50 ± 4.55	4.64 ± 4.75	9.29 ± 5.85	5.57 ± 4.90	4.54 ± 2.80	**3.93** ** ± 3.29**
*ay*	3.86 ± 4.11	4.29 ± 3.75	5.07 ± 4.68	8.21 ± 5.06	12.79 ± 5.96	11.00 ± 6.03	**3.21** ** ± 2.53**	5.71 ± 3.94
Average	13.47 ± 5.18	12.33 ± 5.30	12.07 ± 5.51	10.94 ± 5.13	14.14 ± 5.57	12.56 ± 6.07	10.31 ± 4.85	**8.55** ** ± 4.35**

The lowest error rates for each of the subjects are indicated in bold

**Table 2:** Error rate (%) of proposed SPECTRA predictor and competing methods for dataset 2

Subject	CSP	CSSP	FBCSP	DFBCSP	SFBCSP	SBLFB	CSP-TSM	SPECTRA (Proposed)
*a*	13.20 ± 8.07	13.65 ± 8.19	19.10 ± 9.35	16.80 ± 7.81	17.40 ± 5.93	19.10 ± 9.73	**11.98** ** ± 6.64**	13.00 ± 8.05
*b*	42.80 ± 12.25	42.70 ± 11.38	44.70 ± 11.27	42.90 ± 9.75	45.30 ± 6.59	41.50 ± 11.12	**40.95** ** ± 10.32**	42.50 ± 8.28
*c*	43.70 ± 11.24	39.95 ± 10.21	35.70 ± 9.58	35.20 ± 8.51	43.00 ± 11.62	33.20 ± 12.53	32.16 ± 8.68	**27.90** ** ± 11.53**
*d*	22.40 ± 8.82	14.60 ± 8.75	22.20 ± 8.99	23.50 ± 8.41	29.50 ± 10.13	11.50 ± 7.91	15.74 ± 7.89	13.53 ± 6.12
*e*	18.00 ± 9.74	18.05 ± 9.18	14.00 ± 9.15	18.30 ± 8.84	24.70 ± 10.34	11.60 ± 6.88	9.85 ± 5.77	**9.50** ** ± 8.13**
*f*	22.50 ± 10.84	18.55 ± 8.39	19.60 ± 8.56	14.30 ± 8.57	20.90 ± 6.45	21.20 ± 11.98	14.09 ± 7.13	**13.20** ** ± 6.94**
*g*	7.10 ± 5.06	6.35 ± 4.92	6.90 ± 6.62	9.00 ± 5.05	9.70 ± 4.97	5.90 ± 5.41	7.86 ± 5.67	**5.67** ** ± 4.69**
Average	24.24 ± 9.43	21.98 ± 8.72	23.17 ± 9.07	22.86 ± 8.13	27.21 ± 8.00	20.57 ± 9.36	18.94 ± 7.44	**17.90** ** ± 7.68**

The lowest error rates for each of the subjects are indicated in bold

**Table 3:** Error rate (%) of proposed SPECTRA predictor and competing methods for dataset 3

Subject	CSP	CSSP	FBCSP	DFBCSP	SFBCSP	SBLFB	CSP-TSM	SPECTRA (Proposed)
*B0103T*	23.19 ± 10.14	25.31 ± 9.99	**19.00** ± 8.47	23.25 ± 11.23	26.50 ± 9.24	25.25 ± 10.33	22.50 ± 9.44	22.23 ± 12.03
*B0203T*	41.94 ± 11.04	42.94 ± 11.74	45.63 ± 11.93	40.76 ± 12.45	42.75 ± 12.84	40.75 ± 11.99	39.50 ± 10.43	41.02 ± 11.69
*B0303T*	46.69 ± 9.38	48.44 ± 10.82	49.13 ± 13.54	50.50 ± 12.87	44.97 ± 11.65	50.68 ± 13.34	49.06 ± 11.09	47.36 ± 11.97
*B0403T*	0.75 ± 2.04	0.63 ± 0.60	1.75 ± 1.61	0.75 ± 0.69	**0.38** ** ± 0.35**	0.88 ± 0.73	0.75 ± 2.23	1.46 ± 2.69
*B0503T*	17.85 ± 8.70	42.25 ± 16.33	28.50 ± 8.85	25.00 ± 10.71	25.02 ± 7.38	20.21 ± 10.26	17.18 ± 10.25	**13.78** ** ± 10.51**
*B0603T*	35.19 ± 11.05	23.81 ± 10.94	24.38 ± 9.80	20.88 ± 10.38	20.06 ± 10.70	25.12 ± 12.32	23.01 ± 9.35	**19.31** ** ± 9.44**
*B0703T*	14.50 ± 8.56	13.81 ± 8.11	15.50 ± 6.83	12.13 ± 9.05	12.25 ± 7.47	11.88 ± 9.39	13.81 ± 7.89	**11.67** ** ± 6.71**
*B0803T*	13.06 ± 8.43	14.50 ± 8.56	18.88 ± 11.68	11.13 ± 6.95	12.38 ± 7.63	11.13 ± 8.95	11.44 ± 7.95	**9.79** ** ± 6.29**
*B0903T*	19.13 ± 9.96	17.25 ± 8.66	20.88 ± 10.07	22.25 ± 10.80	25.00 ± 9.62	19.38 ± 10.58	19.75 ± 9.47	**15.63** ** ± 8.65**
Average	23.59 ± 8.81	25.44 ± 9.67	24.85 ± 9.39	22.96 ± 9.61	23.26 ± 8.67	22.81 ± 9.97	21.89 ± 8.67	**20.26** ** ± 8.89**

The lowest error rates for each of the subjects are indicated in bold

The error rates of the proposed scheme compared to other competing methods for dataset 1, dataset 2 and dataset 3 are given in Tables 1, 2 and 3, respectively. The results from Tables 1, 2, and 3 shows that the proposed scheme yields the best results obtaining the lowest average error rates on all three datasets. The proposed scheme shows an improvement in the average error rates (improvement of 1.76% for datasets 1, 1.04% for dataset 2 and 1.63% for dataset 3) compared to the previously best performing CSP-TSM algorithm. An improvement in error rates of 4.92% for datasets 1, 6.34% for dataset 2 and 3.33% for dataset 3 are shown compared to the conventional CSP approach. Considering the performance of the individual subjects, 2 out of 5 subjects for dataset 1, 4 out of 7 subjects for dataset 2 and 5 out of 9 subjects for dataset 3 achieved the lowest error rates using the proposed SPECTRA predictor. Overall, 15 out of 21 subjects showed improvement in performance compared to the CSP-TSM approach with subject “*aa”* of dataset 1 showing highest decrease in error rate (6.43%). Out of these 15 subjects, 13 subjects showed greater than 1% reduction in the error rate. This indicates the advantage of our proposed SPECTRA predictor in comparison to the CSP-TSM approach. It should also be noted that for 4 subjects out of all the subjects used in the evaluation, the error rates increased using the proposed SPECTRA predictor when compared to the CSP-TSM approach with highest increase being 2.50% for the subject “*ay*” of dataset 1. This may be improved or overcome by incorporating automatic selection of the parameter *n* that is subject dependent and will be explored in future works. Our proposed predictor also performed well compared to the TFPO-CSP [51] approach that was evaluated using dataset 1 (achieved error rate of 10.19%) and dataset 2 (achieved error rate of 20.63%).

To add on, the authors of the SBLFB approach have used linear discriminant analysis (LDA) as the classifier. We have used SVM classifier to make a fair comparison between the different methods. It should be noted that the SBLFB approach achieved a slightly better error rate of 11.89% on dataset 1 when the LDA classifier was employed. Furthermore, the authors in [65] proposed an iterative spatio-spectral patterns learning (ISSPL) approach. They evaluated their method using dataset 1 obtaining an average error rate of 5.79%. However, they used a window size of 3.5 s for extracting the trials and thus it cannot be compared with our method as more data is being used by ISSPL approach. Similarly, a cross-correlation based logistic regression (CC-LR) [66] method achieved an average error rate of 6.09% on dataset 1. However, they only used the training data from the competition and evaluated their method using threefold cross-validation. Thus, we cannot compare this method with SPECTRA. In [67], the authors proposed using multiscale principal component analysis for de-noising the EEG signal and extracted higher-order statistics features from wavelet packet decomposition sub-bands. The method was also evaluated using dataset 1, achieving an average error rate of 7.2%. However, it also used 3.5 s window for extracting the trials and hence cannot be directly compared with our approach. In future, we will explore the effect of using multiscale principal component analysis for de-noising the EEG signal with our proposed approach. Also, we will explore the effect of other feature extraction approaches [68–70] and deep learning methods [71] with our current work.

Furthermore, to validate the reliability of the results that has been achieved, Cohen’s kappa coefficient κ is used. Tables 4, 5 and 6 shows the κ values obtained using each of the methods for dataset 1, dataset 2 and dataset 3, respectively. It can be seen from Table 8 (in methods section) that a higher value of κ means a greater strength of agreement. A higher strength of agreement means that the results are more reliable. Our proposed scheme attained the best average κ values for all the 3 datasets. This shows that the results of the proposed scheme are more reliable when compared with other competing methods. Considering the average κ values, a very good strength of the agreement of classes for dataset 1 and good prediction of classes for dataset 2 and dataset 3 have been achieved. It can be noted that the κ values for some subjects (such as subject “*av”* of dataset 1, subjects “*b”* and “*c”* of dataset 2 and subjects “*B0203T”* and “*B0303T”* of dataset 3) are very low. These results are consistent with the results of other methods and may be mainly due to low quality signals being recorded, which are contaminated by noise. Considering individual subjects 4 out of 5 for dataset 1, 5 out of 7 for dataset 2 and 6 out of 9 subjects for dataset 3 achieved good or very good strength of agreement using the proposed scheme. While on the other hand, 3 out of 5 subjects for dataset 1, 6 out of 7 subjects for dataset 2 and 5 out of 9 subjects for dataset 3 attained the best κ values using the proposed scheme.

**Table 4:** Cohen’s kappa coefficient values of proposed and competing methods for dataset 1

Subject	CSP	CSSP	FBCSP	DFBCSP	SFBCSP	SBLFB	CSP-TSM	SPECTRA
*aa*	0.613	0.659	0.601	**0.816**	0.394	0.664	0.636	0.793
*al*	0.927	0.940	0.970	0.976	0.917	0.973	0.950	**0.979**
*av*	0.426	0.423	0.384	0.329	0.389	0.439	0.543	**0.567**
*aw*	0.800	0.837	0.837	0.906	0.743	0.889	0.896	**0.921**
*ay*	0.903	0.926	0.881	0.847	0.763	0.780	**0.950**	0.886
Average	0.734	0.757	0.735	0.775	0.641	0.749	0.795	**0.829**

The best values for each of the subjects are highlighted in bold

**Table 5:** Cohen’s kappa coefficient values of proposed and competing methods for dataset 2

Subject	CSP	CSSP	FBCSP	DFBCSP	SFBCSP	SBLFB	CSP-TSM	SPECTRA
*a*	0.736	0.727	0.618	0.664	0.652	0.618	**0.757**	0.730
*b*	0.144	0.146	0.106	0.142	0.094	0.170	0.206	0.150
*c*	0.126	0.201	0.286	0.290	0.140	0.336	0.359	**0.418**
*d*	0.552	0.708	0.556	0.530	0.410	0.770	0.696	**0.733**
*e*	0.640	0.639	0.720	0.634	0.506	0.768	0.826	**0.807**
*f*	0.550	0.629	0.608	0.714	0.582	0.576	0.717	**0.777**
*g*	0.858	0.873	0.862	0.820	0.806	0.882	0.851	**0.887**
Average	0.515	0.560	0.537	0.542	0.456	0.589	0.630	**0.643**

The best values for each of the subjects are highlighted in bold

**Table 6:** Cohen’s kappa coefficient values of proposed and competing methods for dataset 3

Subject	CSP	CSSP	FBCSP	DFBCSP	SFBCSP	SBLFB	CSP-TSM	SPECTRA
*B0103T*	0.536	0.494	**0.620**	0.535	0.470	0.495	0.550	0.554
*B0203T*	0.161	0.141	0.088	0.185	0.145	0.185	**0.210**	0.179
*B0303T*	**0.106**	0.031	0.018	0.010	0.100	0.014	0.010	0.054
*B0403T*	0.983	0.988	0.965	0.985	**0.993**	0.983	0.985	0.971
*B0503T*	0.650	0.115	0.430	0.500	0.499	0.595	0.655	**0.725**
*B0603T*	0.296	0.524	0.513	0.583	0.598	0.457	0.530	**0.614**
*B0703T*	0.710	0.724	0.690	0.758	0.755	0.763	0.724	**0.767**
*B0803T*	0.739	0.710	0.623	0.778	0.753	0.776	0.771	**0.804**
*B0903T*	0.618	0.655	0.583	0.555	0.500	0.613	0.605	**0.688**
Average	0.534	0.487	0.503	0.543	0.535	0.542	0.560	**0.595**

The best values for each of the subjects are highlighted in bold

In this work, we have used a single wide band to keep the computational complexity of the proposed method low as using multiple sub-bands would result in an increased computational complexity. However, using multiple sub-bands may further improve the performance of the system and will be studied in future. Table 7 shows the time taken to process and classify a MI EEG signal for different methods (Matlab running on a personal computer at 2.4 GHz (Intel(R) Core(TM) i5) has been used for all processing). Our proposed SPECTRA predictor takes 6.10 ms to process and classify a trial of EEG signal. Thus, the proposed scheme is suitable for real-time applications and is computationally efficient for portable devices. Our proposed approach also takes less time to process and classify a trial compared to other competing methods such as DFBCSP, SFBCSP and SBLFB. SPECTRA takes more time to process and classify a trial compared to CSP, CSSP and CSP-TSM as it employs these approaches.

**Table 7:** Test time required by different algorithms for single-trial MI EEG signal classification

Method	CSP	CSSP	FBCSP	DFBCSP	SFBCSP	SBLFB	CSP-TSM	SPECTRA
Time (ms)	2.30	4.30	14.22	10.80	19.86	13.10	2.60	6.10

## Discussion

**Fig. 1:**
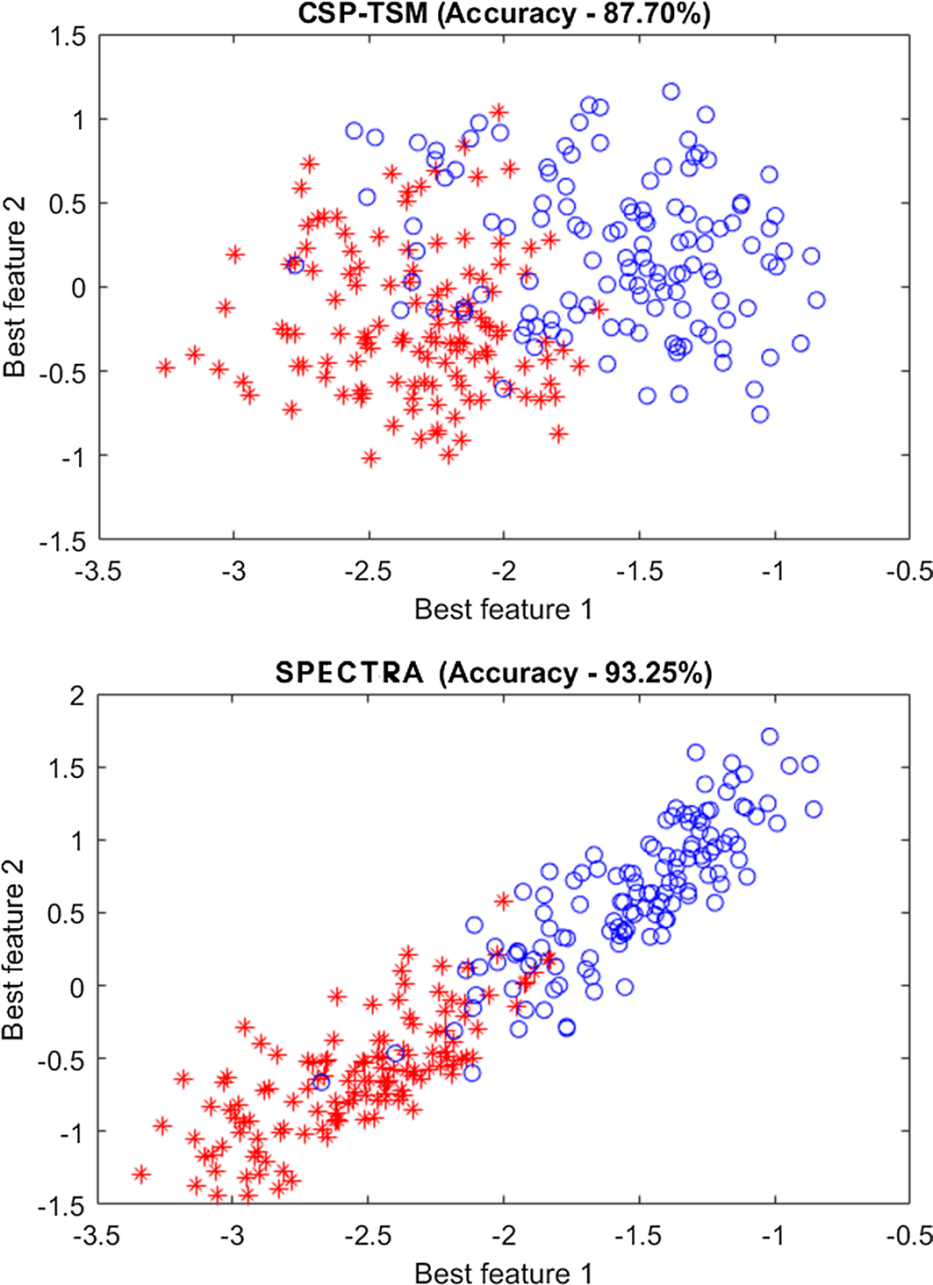
Distribution of the two most significant features obtained by CSP-TSM, and proposed SPECTRA predictor, respectively, using subject *aa* of dataset 1

In this study, we have performed feature selection using F-score in order to remove redundant features so that only significant features are used. Top *r* = 10 features has been selected [64]. Figure 1 shows the feature distribution of the top two features for CSP-TSM and the proposed scheme. It can be seen that using the proposed scheme effectively finds more separable features that accounts for the improved performance and usefulness of the proposed system.

Furthermore, as mentioned before, we have only used dataset 2 for selecting the parameter *n*. This has been done so that we do not have to tune the parameters for each new dataset that is used and so that the parameters selected can perform well on all datasets. This will reduce the training time by not having to perform parameter selection on other datasets. It is seen that the parameters selected for the proposed method in this work performed well as promising results have been obtained for all the three datasets.

To show the significance of the proposed method, we have performed paired t-test with 1% significance level. The average individual error rate results of the proposed scheme have been compared with that of the 2nd best method (CSP-TSM). The p-value obtained was 0.0036, which shows that significant improvements are achieved by the proposed scheme.

**Fig. 2:**
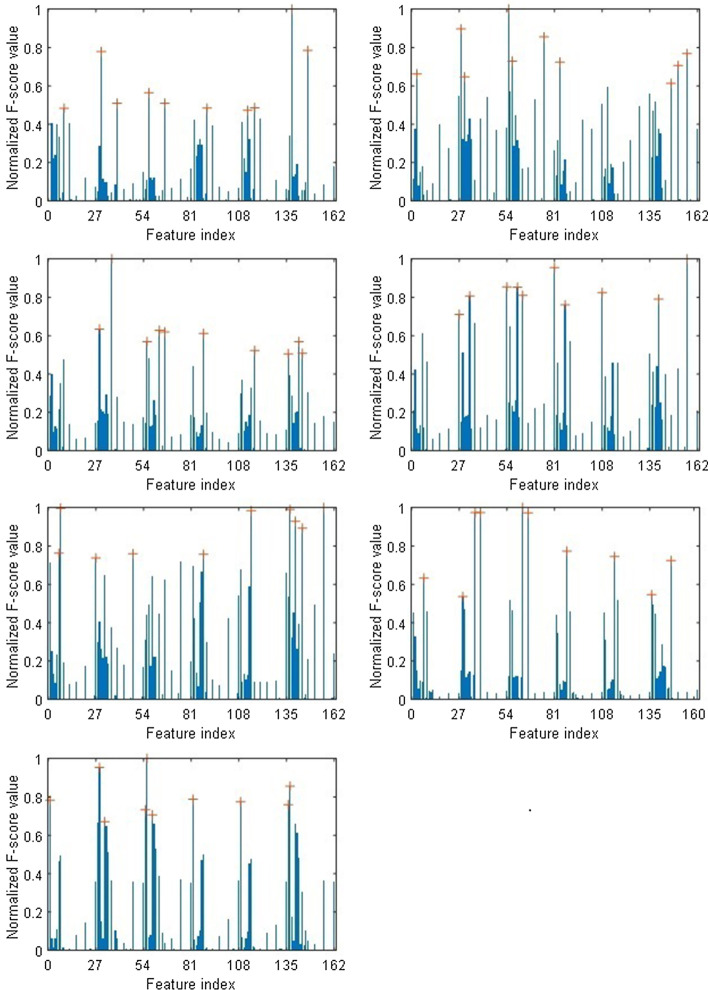
Normalized F-score feature rankings for subjects *a* to *g* of dataset 2. The top 10 features are indicated with ‘ + ’ sign

**Fig. 3:**
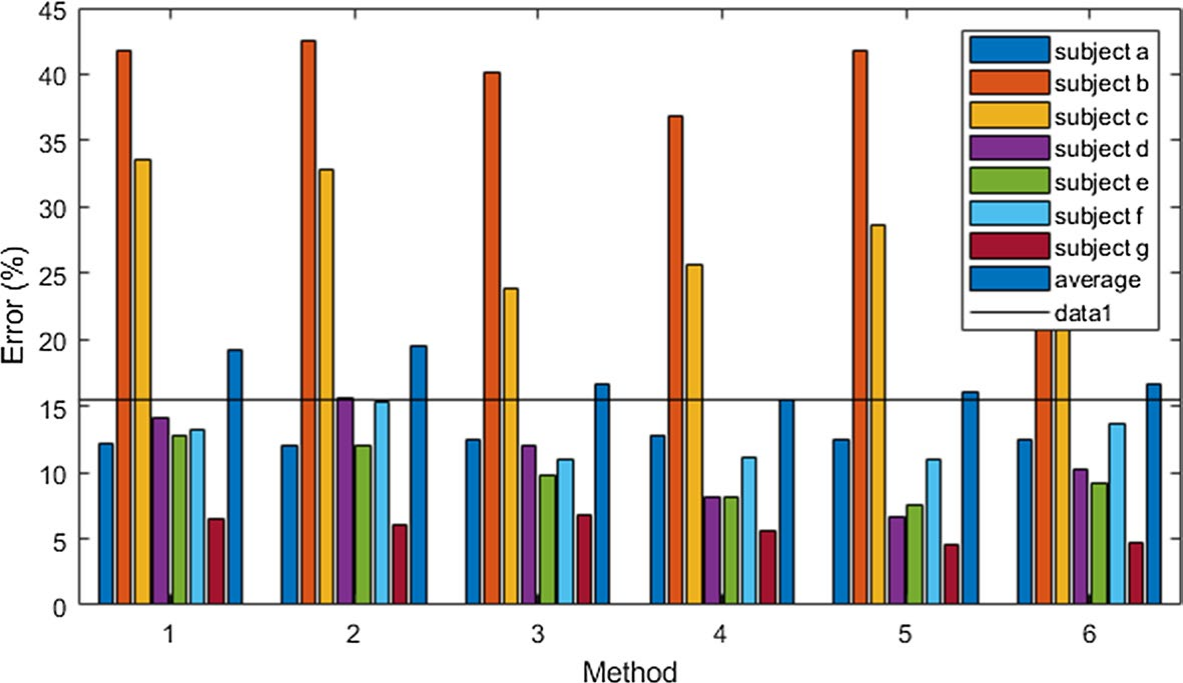
Error rates of different frameworks using dataset 2. Method 1 and 2 refers to results of experiments 1 and 2 respectively. Methods 3–6 represent the proposed SPECTRA predictor using top 5, 10, 15, and 20 selected features, respectively

**Fig. 4:**
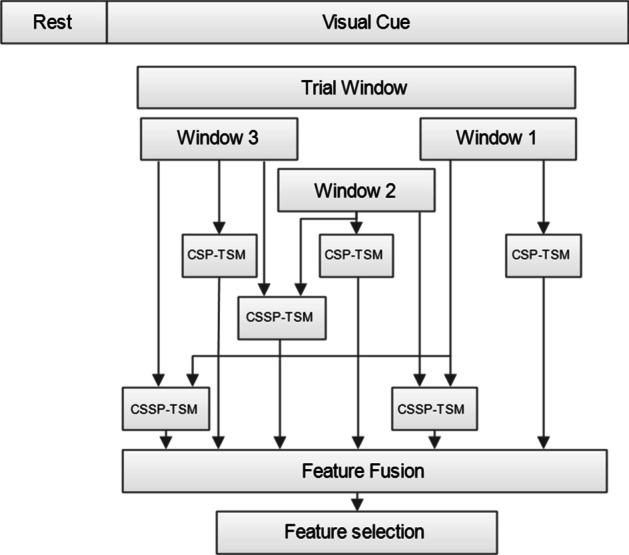
The framework of the proposed SPECTRA predictor

Moreover, there are various ways of combining the temporal windows for CSSP-TSM approach as this can be done by simply using only two temporal windows. Figure 2 shows the normalized F-score ranking of the features for the subjects of dataset 2. The number of features obtained by each CSP-TSM or CSSP-TSM process is 27 (6 CSP features and 21 TSM features), therefore a total of 162 (6 × 27) features are obtained. CSP-TSM and CSSP-TSM processes refer to the blocks performing CSP-TSM and CSSP-TSM, as shown in Fig. 1, having 3 CSP-TSM and 3 CSSP-TSM processes. The output of each of these processes is a combination of CSP and TSM features. It can be seen from Fig. 2 that all the CSP-TSM and CSSP-TSM processes give more separable features. Hence the framework given in Fig. 1 has been adopted. In this work we are performing feature selection rather than selecting several CSP-TSM and CSSP-TSM processes only. This has been proposed after evaluating different frameworks. We have evaluated selecting only features of several top *k* CSP-TSM or CSSP-TSM processes from the 6 processes shown in Fig. 4 (refer to methods section). To select these top *k* CSP-TSM or CSSP-TSM processes, we again used the F-score. Two experiments were conducted for this. Experiment 1 used individual F-score feature ranking to select top *k* CSP-TSM or CSP-TSSM processes i.e. the top *k* CSP-TSM or CSSP-TSM processes with highest individual feature rankings were selected. In experiment 2, the average of the F-score feature rankings of all features of each CSP-TSM and CSSP-TSM processes were used to select the top k CSP-TSM or CSSP-TSM processes. We have used *k* = 4 (similar to the band selection procedure in [61]) for experiments 1 and 2. It is evident from Fig. 3 that our proposed scheme with top 10 features selected gives the best result.

To add on, the results of BCI Competition were obtained using specific test data only (which was specifically for BCI competition only). Cross-validation using all the data is a more effective way to test a model’s performance and has been mostly utilized to compare the different methods proposed for BCI applications. This is the reason why the results of BCI Competition have not been compared with our work as done by other researchers. Moreover, as mentioned earlier, the value of parameter *n* selected did not produce the optimal results for all individual subjects and will be investigated in future works. We will also consider other feature extraction, feature selection and classifiers [72] for future works.

Convolutional neural network (CNN) has gained a lot of attention over the recent years. Therefore, in future, we will evaluate the use of CNN for MI-EEG signal recognition by developing hybrid models utilizing CNN with SPECTRA. Furthermore, good performance is noted by CNN on image data, therefore, DeepInsight [71] will be used to transform the EEG signal to image before being fed as input the CNN model. Long short-term memory network has also performed well for MI-EEG signal recognition [73] and we will also consider using LSTM network to further improve the performance of the proposed SPECTRA predictor.

## Conclusions

In this work, we have utilised the CSP-TSM approach with multiple temporal delayed windows for extracting more separable features, using CSP and CSSP methods. Parameters such as the temporal delay and number of windows have been optimized. F-score for feature selection is proposed over Lasso that is used by the CSP-TSM approach due to its reliability and enhanced ability in selecting significant features. Our proposed scheme out-performed other competing approaches and achieved the lowest average error rates and highest average Cohen’s kappa coefficient values. A fixed wide band has been used for all evaluation. Developing sophisticated algorithms which will automatically learn filter bands that will give optimal performance for each subject may further improve the performance of the proposed system. Our proposed scheme can be potentially used for the development of improved and computationally efficient BCI systems.

## Methods

### Public benchmark datasets

We have evaluated the performance of the proposed scheme using 3 datasets that are publicly available: BCI Competition III dataset IVa [74], BCI Competition IV dataset I [75] and, BCI Competition IV dataset IIb [75] referred to as dataset 1, dataset 2 and dataset 3 from here onwards, respectively.

All the three datasets contain two class MI tasks. The EEG signals of right hand and left foot MI tasks recorded from five subjects using 118 channels of EEG signals is contained in dataset 1. The signals sampled at 100 Hz are used with each subject having 140 trials for each task. Dataset 2 contains MI EEG signals of seven subjects recorded using 59 channels at 1000 Hz. The down sampled data at 100 Hz is used and it contains 200 trials for each subject containing almost equal number of trials for each MI tasks. Dataset 3 contains EEG signals of nine subjects. It contains 3 channels right hand and left hand MI tasks sampled at 250 Hz. As used in [62], we have only used data from session three for evaluation. Each subject contains 80 trials of each MI task. For a complete explanation of the datasets, refer to http://www.bbci.de/competition/.

### CSP feature extraction

CSP has become one of the most popular and widely used techniques for feature extraction of MI EEG signals. Spatial filters *W*_*csp*_ are learned by the CSP algorithm, which maximizes the variance of one class while minimizing the variance of the other class. This offers an effective method to approximate the discerning information of the MI tasks. Given an EEG signal *X*_*i*_ $\in R^{C \times T}$ where *i* denotes the *i*-th trial, c denotes the number of channels data contained by the EEG signal and t is the number of sample points. The learned spatial filters are used to transform the EEG signal to a new time series using (1).

1 $$Z_{i} = W_{CSP}^{T} X_{i}$$

The variance based CSP features are then extracted from the spatially transformed signal $Z_{i}$ using (2), where $f_{i}^{k}$ is the *k-*th feature of the *i-*th trial and var($Z_{i}^{j}$) denotes the variance of *j-*th row of $Z_{i}$. Refer to [76] for a detailed description of the CSP algorithm.

2 $$f_{i}^{k} = \log \left( {\frac{{{\text{var}} (Z_{i}^{k} )}}{{\mathop \sum \nolimits_{j = 1}^{2m} {\text{var}} (Z_{i}^{j} )}}} \right)$$

### CSP-TSM feature extraction

The CSP-TSM approach has been proposed for extracting significant tangent space features while keeping the computational complexity low [52]. The concept of Riemannian geometry is utilized by the CSP-TSM approach. The normalized covariance matrix ${\varvec{\varSigma}}_{{\varvec{i}}}$ of each of the spatially filtered trial ${\varvec{Z}}_{{\varvec{i}}}$ is calculated. The Riemannian distance ${\varvec{\delta}}_{{\varvec{R}}}$ is then computed using (3), where ${\varvec{\varSigma}}$ is the Riemannian mean of all the trial covariance matrices ${\varvec{\varSigma}}_{{\varvec{i}}}$ (from the training set) and is calculated using (4), the logarithmic mapping ${\text{Log}}_{{\varvec{\varSigma}}} \left( {{\varvec{\varSigma}}_{{\varvec{i}}} } \right)$ is given by (5) and ${\varvec{s}}_{{\varvec{i}}}$ represents the normalized tangent space vector (also referred to as tangent space features). The upper(·) in (3) means vectorizing the upper triangular portion of the symmetric matrix and multiplying the out-of-diagonal elements [77] by $\sqrt {\mathbf{2}}$.

3 $$\delta_{R} \left( {\Sigma ,\Sigma_{i} } \right) = \left| {\left| {{\text{Log}}_{\Sigma } \left( {\Sigma_{i} } \right)} \right||_{\Sigma}}= \right||{\text{upper}}\left( {{\Sigma }^{ - 1/2} {\text{Log}}_{\Sigma } \left( {\Sigma_{i} } \right)\Sigma^{ - 1/2} } \right)\left| {|_{2}}= \right|\left| {s_{i} } \right||_{2}$$

4 $$\sum = R\left( {\Sigma_{i} } \right) = \mathop {argmin}\limits_{\Sigma \in \Sigma \left( n \right)} \mathop \sum \limits_{i = 1}^{N} \delta_{R}^{2} \left( {\Sigma ,\Sigma_{i} } \right)$$

5 $${\text{Log}}_{\Sigma } \left( {\Sigma_{i} } \right) = \Sigma^{1/2} \log (\Sigma^{ - 1/2} \Sigma_{i} \Sigma^{ - 1/2} ) \Sigma^{1/2}$$

The above process maps all the trial covariance matrices $\Sigma_{i}$ into the tangent space. Thus, the features obtained from tangent space mapping are fused together with the CSP features and significant features are selected. The selected features are then used for classification. A complete description of the CSP-TSM approach can be obtained from our preceding work [64].

### Proposed approach

In this study, we propose an effective subject-dependent method of feature extraction by utilizing the CSP-TSM approach. The general conceptual framework of the proposed methodology for obtaining significant features is shown in Fig. 4. Usually, only a single window of 2.0–3.0 s is used for MI-based BCI applications. Here, we propose to use *n* multiple temporal delayed windows in two different ways. Firstly, the variance based CSP features and TSM features are computed for each of the *n* = 3 windows (the choice of *n* used is explained in the following sub-section). Secondly, the CSSP approach is utilized for extracting further information. CSSP method involves inserting a temporal delayed window to the trial signal and performing CSP on this modified trial signal that is obtained. The CSSP approach was proposed for improving the performance of CSP. The time delay value $\tau$ influences the performance of the system and needs to be chosen carefully. In this work, the time delay ($\tau$ sample points) has been selected using the cross-validation technique. All combinations of the *n* windows are used for obtaining new CSSP trial windows given by (6), where $W_{i}$ is the *i*-th window of the original signal (refer to Fig. 4), $W_{CSSP}^{i,i + j}$ is the signal obtained by inserting the $W_{i + j}$ temporal delayed window to window $W_{i}$ and $i = 1:n - 1$. CSP variance-based features and TSM features are attained from the windows obtained from (6).

6 $$W_{CSSP}^{i,i + j} = \left[ {\begin{array}{*{20}c} {W_{i} } \\ {W_{i + j} } \\ \end{array} } \right]{ };j = 1:n - i$$

All the features obtained are fused together to form the feature vector. Using (7) the F-score ranking of the features is then computed, where $\overline{F}_{i}$ is average value of the *i*-th feature, $\overline{{F_{i}^{ + } }}$ and $\overline{{F_{i}^{ - } }}$ are the average values of the *i*-th feature for the positive and negative samples respectively, $N^{ + }$ and $N^{ - }$ refers to the total number of positive and negative samples respectively and $F_{k,i}$ refers to the *k*-th sample of the *i*-th feature. The positive samples for all the three datasets were right-hand MI task samples while the negative samples were left-foot MI task samples for dataset 1 and 2 and left hand MI task samples for dataset 3. The F-score values obtained are arranged in descending order and the top *r* features are selected, which are classified using support vector machine (SVM) classifier.

7 $$F_{score} \left( i \right) = \frac{{\left( {\overline{{F_{i}^{ + } }} - \overline{F}_{i} } \right)^{ 2} + \left( {\overline{{F_{i}^{ - } }} - \overline{F}_{i} } \right)^{ 2} }}{{\frac{1}{{N^{ + } - 1}}\mathop \sum \nolimits_{k = 1}^{{N^{ + } }} \left( {F_{k,i}^{ + } - \overline{{F_{i}^{ + } }} } \right)^{ 2} + \frac{1}{{N^{ - } - 1}}\mathop \sum \nolimits_{k = 1}^{{N^{ - } }} \left( {F_{k,i}^{ - } - \overline{{F_{i}^{ - } }} } \right)^{ 2} }}$$

SVM is a supervised learning technique and has been effectively used for both regression and classification problems. A hyperplane that maximizes the separation of the support vectors is determined by the SVM algorithm. In this study we employed an SVM classifier having radial basis kernel function. The use of kernel function allows non-linear data to be mapped to a linearly separable higher dimensional plane.

### Parameter selection

**Fig. 5:**
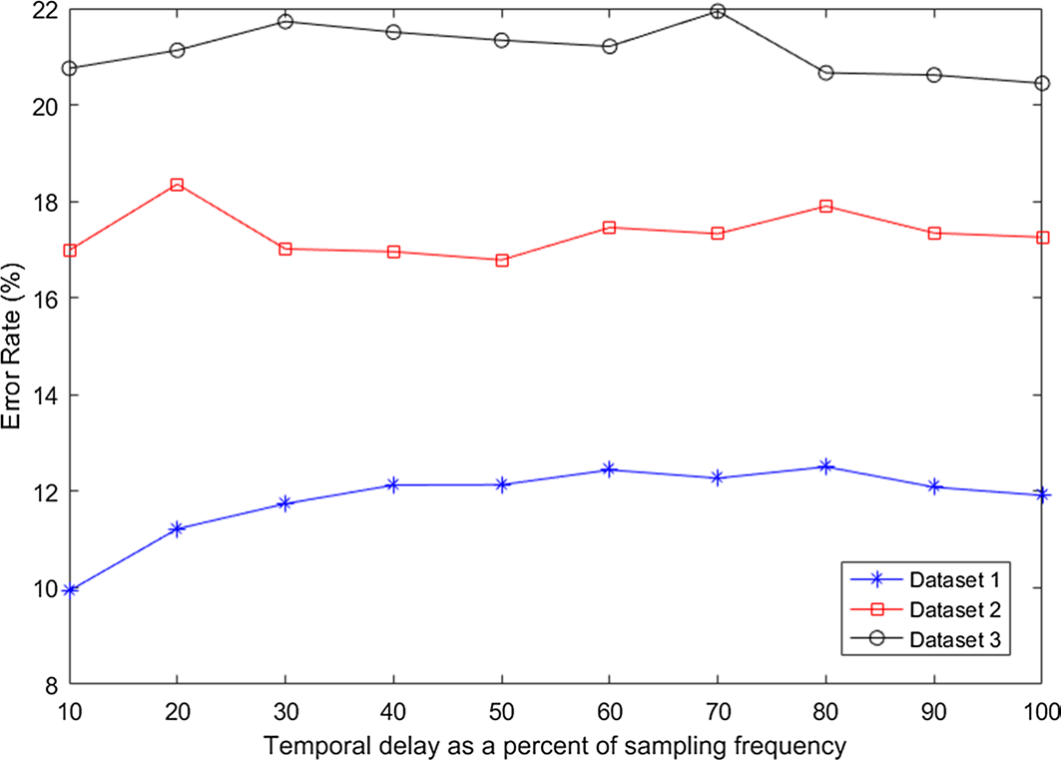
Average error rates for different values of temporal delay as a percentage of sampling frequency

**Fig. 6:**
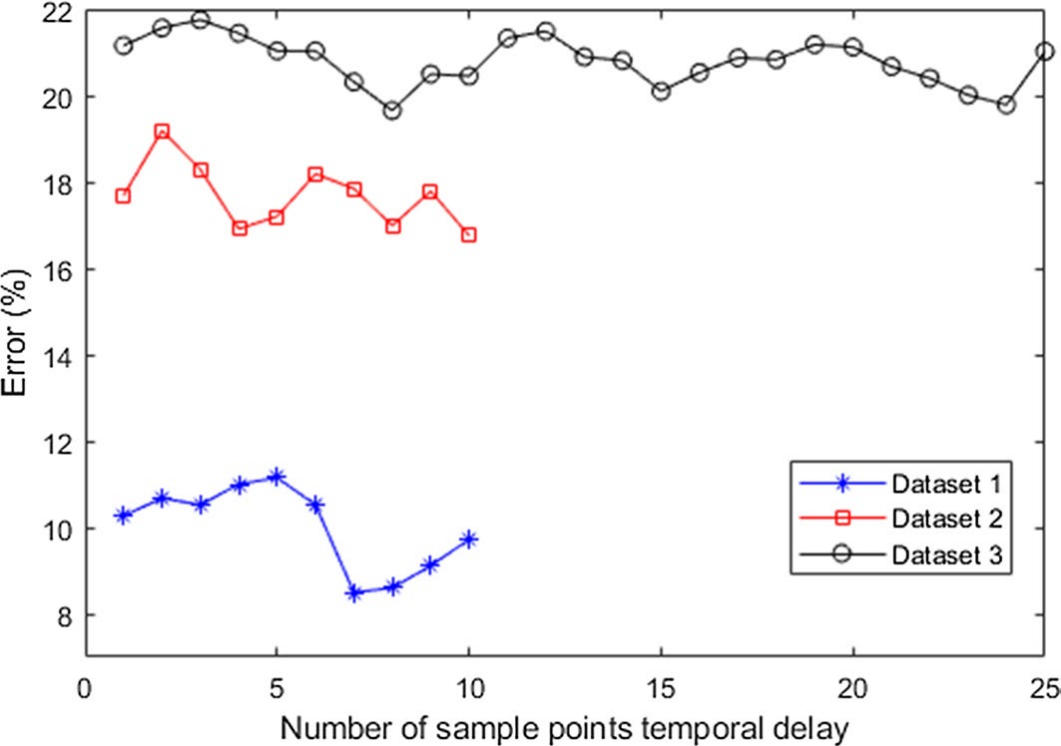
Average error rates for different values of temporal delay

Multiple temporal delayed windows have been utilized in this study. Two factors are of importance in this process: window size and temporal time delay $\tau$ between windows. Different subjects have different response rate to the onset cue. Therefore, determining the exact location of the MI task in the EEG signals needs to be investigated and clustering methods [78–80] can be utilised for this purpose. We have fixed the window size to 2.0 s in our work, the same as used by most of the researchers [34, 48, 58, 62]. To determine the best $\tau$ value that would yield the optimal performance, we have conducted the following experiments. Firstly, the $\tau$ value was varied from 10 to 100% of the sampling frequency for each of the datasets and the results are shown in Fig. 5. In selecting the $\tau$ value, it is very important to consider real time BCI applications. Considering that real time BCI applications will also be portable, the computational complexity should be kept to a minimum. Therefore, it is desirable to select the smallest $\tau$ value that will produce near to optimal results. From Fig. 5, it can be seen that using 10% of sampling frequency as the $\tau$ value gives near optimal performance for all three datasets. To further refine the $\tau$ parameter (since now it is clear that using larger $\tau$ values would not improve the performance), $\tau$ values from 1 to 10% of sampling frequency were evaluated (results shown in Fig. 6). It can be noted from Fig. 6 that for dataset 1 and dataset 2 only 10 (10% of 100) sample points are shown whereas for dataset 3, 25 (10% of 250) sample points are shown due to the signals being sampled at different frequencies. It can also be noted from Fig. 6 that optimal performance is obtained for different subjects at different $\tau$ values. Thus, another tenfold cross-validation has been performed on the training data (which is obtained from the initial tenfold cross-validation) for the selection of subject-dependent $\tau$ values that will give optimal performance. In this way, the test samples are not used during parameter tuning.

**Fig. 7:**
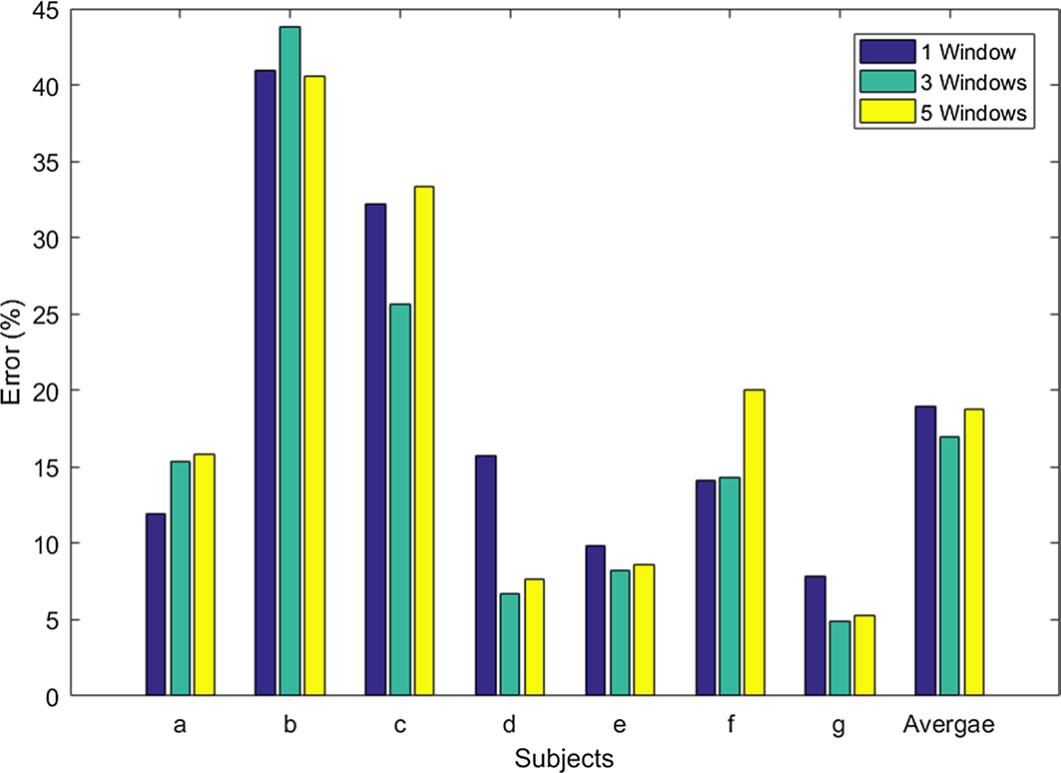
Error rates for using different number of windows (for dataset 2)

The other parameter that needed to be selected was *n*, the number of windows. We have evaluated *n* = [1, 3, 5] and the results are shown in Fig. 7. We have randomly selected dataset 2 for selecting the parameter *n*. Using only 1 window will result in the CSP-TSM approach. It is evident from Fig. 7 that using a high number of windows did not enhance the system performance and would increase the computational complexity of the system. All subjects except subjects *a* and *b* performed well using 3 windows compared to using 1 or 5 windows. Therefore, to retain a low computation complexity of the proposed scheme while also producing optimal performance, we have chosen *n* = 3.

**Fig. 8:**
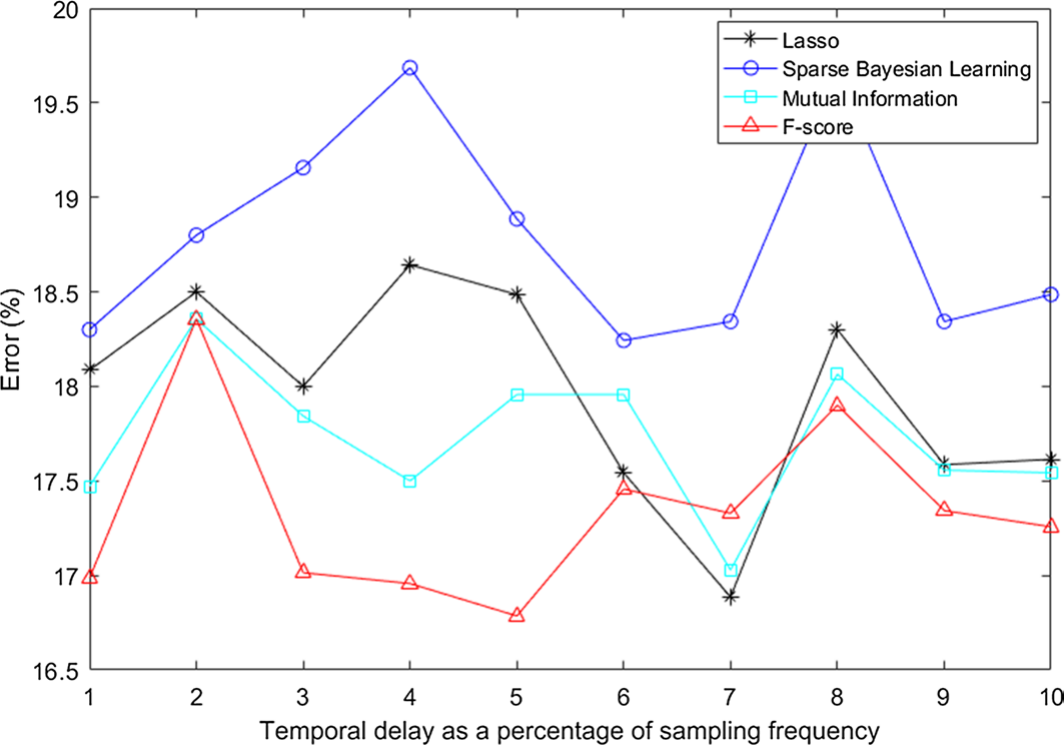
Error obtained for different feature selection algorithms using dataset 2

We have also evaluated four different feature selection algorithms (Lasso [52, 81], sparse Bayesian learning [42], mutual information [9] and F-score based feature selection algorithms) in order to choose the best performing algorithm. Figure 8 shows the error rates obtained for different feature selection algorithms using dataset 2. It can be noted that using F-score yields the minimum error rates for almost all temporal delay values showing that it is a robust and reliable feature selection method. This is the reason why we have used F-score in this work for feature selection instead of the Lasso method as used in CSP-TSM approach.

### Performance measures

To appropriately rank and compare our proposed scheme with competing methods, two performance measures, error rate and Cohen’s kappa coefficient (κ) have been used. Error rate is a commonly used measure for evaluating the performance of BCI systems, which shows the percentage of trials that are classified incorrectly. κ is utilised for validating the reliability of the results which statistically accesses the consistency of agreement among two classes. κ is calculated using (8), where $p_{e}$ is the chance of agreement (in percentage) that is expected and $p_{a}$ is the actual agreement (in percentage). Table 8 shows the strength of agreement for different κ values [82].

8 $${\upkappa } = \frac{{p_{a} - p_{e} }}{{1 - p_{e} }}$$

**Table 8:** Strength of agreement for different Cohen’s kappa coefficient values

κ	< 0.20	0.21–0.40	0.41–0.60	0.61–0.80	0.81–1.0
Strength	Poor	Fair	Moderate	Good	Very Good

## Data Availability

The datasets used in this study are publicly available at http://www.bbci.de/competition.

## Abbreviations

BCI: Brain computer interface
CSP: Common spatial pattern
CSSP: Common spatio-spectral pattern
DFBCSP: Discriminant filter bank common spatial pattern
EEG: Electroencephalography
FBCSP: Filter bank CSP
LDA: Linear discriminant analysis
MI: Motor imagery
SBCSP: Sub-band common spatial pattern
SBLFB: Sparse Bayesian learning of filter banks
SFBCSP: Sparse filter bank CSP
SFTOFSRC: Spatial-frequency-temporal optimized feature sparse representation-based classification
SPECTRA: Spatial-frequency-temporal feature extraction
SVM: Support vector machine
TFPO: Temporal filter parameter optimization
